# Impact of a pilot multimodal intervention to decrease antibiotic use for respiratory infections in a geriatric clinic

**DOI:** 10.1017/ash.2021.238

**Published:** 2022-01-10

**Authors:** Lakshmi R. Chauhan, Misha Huang, Mona Abdo, Skotti Church, Danielle Fixen, Samantha MaWhinney, Matthew Miller, Kristine M. Erlandson

**Affiliations:** 1 Division of Infectious Diseases, Department of Medicine, Anschutz Medical Campus, University of Colorado, Aurora, Colorado; 2 Department of Epidemiology, Colorado School of Public Health, Aurora, Colorado; 3 Department of Biostatistics and Informatics, Colorado School of Public Health, Aurora, Colorado; 4 Division of Geriatric Medicine, Department of Medicine, Anschutz Medical Campus, University of Colorado, Aurora, Colorado; 5 Department of Pharmacy, University of Colorado Hospital, Aurora, Colorado

## Abstract

**Background:**

More than 80% of antibiotics are prescribed in the outpatient setting, of which 30% are inappropriate. The National Action Plan for Combating Antimicrobial Resistance called for a 50% decrease in outpatient antibiotic use by 2020. Inappropriate antibiotics are associated with adverse reactions and *Clostridioides difficile* infection, especially among older adults.

**Study design:**

Before and after study.

**Methods:**

We performed a quality improvement initiative at the University of Colorado Seniors Clinic. Providers received education on antibiotic guidelines, electronic antibiotic order sets were introduced with standardized stop dates. Antibiotic use data were collected for 6 months before and 6 months after the intervention, from December to May to avoid seasonal variation. Descriptive statistics and linear mixed-effects regression models were used for this comparison.

**Results:**

Total antibiotic prescriptions for acute respiratory conditions decreased from 137 prescriptions before the intervention (December 1, 2017, to May 31, 2018) to 112 prescriptions after the intervention (December 1, 2018, to May 31, 2019), driven primarily by decreases in antibiotic prescriptions for pneumonia, sinusitis, and bronchitis. Prescriptions for broad-spectrum antibiotics declined following the intervention including decreases in levofloxacin from 12 (9%) to 3 (3%) and amoxicillin-clavulanate from 15 (12%) to 7 (7%). We detected significant reductions in prescribed antibiotic durations (days) after the intervention for sinusitis (estimate, −2.0; 95% CI, −3.1 to −1.0; *P* = .0003), pharyngitis (estimate, −2.5; 95% CI, −4.6 to −0.5; *P* = .018), and otitis (−3.2; 95% CI, −5.2 to −1.3; *P* = .008).

**Conclusions:**

Low-cost interventions were initially successful in changing patterns of antibiotic use and decreasing overall antibiotic prescribing among older patients in the outpatient setting. Long-term follow-up studies are needed to determine the sustainability and clinical impact of these interventions.

Rising antimicrobial resistance is a global public health crisis, resulting in 2.8 million infections and 35,900 deaths yearly.^1^ The availability of limited antibiotic options for resistant organisms poses a threat particularly to vulnerable populations including older adults and immunocompromised patients. Although antibiotic stewardship tends to focus on the hospital setting, outpatient antimicrobial use contributes to >80% of prescribed antibiotics,^2^ and 30% of prescribed outpatient antibiotics are considered inappropriate.^3,4^ The majority of these are for antibiotic prescriptions for upper respiratory conditions. Prior large-scale studies have repeatedly demonstrated that antibiotics are overprescribed for outpatient acute respiratory conditions,^5,6^ and the rate of inappropriate prescriptions ranges from 35% to 76% based on the specific condition.^6–8^

Not only do inappropriate antibiotic prescriptions contribute to an increase in antimicrobial resistance, but antibiotics are also associated with serious adverse effects such as drug–drug interactions, allergic reactions, neurologic or psychiatric effects, and *Clostridiodes difficile* infection.^9^ According to the US Centers for Disease Control and Prevention (CDC), there are 223,900 cases of *C. difficile* infection and >12,800 deaths yearly. Older adults are at higher risk of adverse effects and complications associated with antibiotic prescriptions^10–12^; however, they may also receive prescriptions at a higher rate due to increased concern for severe infections or nonspecific symptoms. In a large cohort of low-risk elderly patients, 46% of patients with nonbacterial acute upper respiratory infection received an antibiotic prescription.^13^

Acknowledging the key role that outpatient antibiotic use plays in fueling antimicrobial resistance, the US National Action Plan called for a decrease in outpatient antimicrobial use by 50% by 2020.^14^ The CDC has formulated guidance for antimicrobial stewardship in the outpatient setting,^15^ but implementing those guidelines and changing patterns of antibiotic use among providers is a challenge. The most effective interventions have used a combination of strategies, incorporating technology (electronic prescribing decision support), personnel support (education, pharmacist intervention), organization (peer comparison, audit and feedback), and patient education (commitment posters, media campaigns).^16–18^

The goals of this study were to implement and evaluate initial effects of multifaceted low-cost interventions to decrease antibiotic use, and we focused on prescriptions within a geriatric clinic. We hypothesized that there would be inappropriate antibiotic selection and duration for common upper respiratory conditions especially bronchitis and sinusitis and that our intervention could decrease duration of prescribed antibiotics and increase prescription concordance with guidelines.

## Methods

We performed a quality improvement initiative at the University of Colorado Seniors Clinic to decrease antibiotic use in acute respiratory conditions. The intervention was targeted toward improving clinician knowledge and providing tools for clinicians to provide nonantibiotic recommendations to patients presenting with acute respiratory symptoms. This protocol was submitted to the Colorado Multiple Institutional Review Board, which did not consider it to be human subject research.

To obtain preintervention data, we collected antibiotic type, duration, and indication for antibiotic prescriptions for acute respiratory tract conditions from December 1, 2017, to May 31, 2018. Data were derived through a newly developed electronic heath record report, incorporating diagnosis codes and prescription data on dose and duration. This report was validated over multiple months by a team of Epic software specialists (Epic, Verona, WI), clinical data managers, and infectious disease physicians. Acute respiratory tract infections included acute sinusitis, acute pharyngitis, acute otitis media, acute bronchitis, pneumonia, and acute exacerbation of chronic obstructive pulmonary disease (COPD).

The interventions performed included 4 components and was introduced in June 2018 in a stepwise design. First, an infectious disease physician (L.C.) attended a meeting with the clinic faculty to provide in-person education on antibiotic guidelines. One geriatric physician (S.C.) and the geriatric pharmacist (D.F.) were coinvestigators on this project and met at regular intervals with the infectious disease faculty throughout the project. The geriatric pharmacist was integrated in the clinic and was available for antibiotic-related questions during clinic hours. Second, in July 2018, we implemented electronic antibiotic order sets for common ambulatory infectious syndromes that were prepopulated with first- and second-line antibiotic choices, indications, and durations, and instructed providers on their availability and usage. Third, beginning in November 2018, patient education posters were displayed in the clinic waiting areas^19^ (Supplementary Images 2 and 3). Finally, in January 2019, we rolled out viral prescription pads, which provided instructions and recommendations for symptomatic relief for a viral syndrome (Supplementary Image 1). Comparison intervention data on antimicrobial prescriptions, indication, and duration were collected from December 1, 2018, to May 31, 2019, to avoid seasonal variation in antibiotic use.

### Statistical analysis

Antibiotic use data before and following all interventions were obtained for analysis. Antibiotic data after individual intervention were not compared because there was a delay in validating our database. Frequency and percent were calculated for categorical variables by indications for pre- and postintervention time points. Median durations and interquartile ranges (IQRs) were calculated for antibiotic use overall and for respiratory indications. Antibiotic use before and after the intervention was compared using linear mixed regression models with provider as a random effect. Estimates, 95% confidence intervals, and *P* values are reported. The duration of antibiotic was set to 5 for missing values for azithromycin, which were part of composite prescription orders. Number of antibiotic prescriptions for individual providers was calculated for pre- and postintervention periods. Significance was defined as *P* < .05 and all statistical analyses were conducted in SAS version 9.4 software (SAS Institute, Cary, NC).

## Results

Prior to the intervention, 362 total antibiotic prescriptions for 263 patients were given during 8,557 clinic visits, accounting for 42.3 antibiotic prescriptions per 1,000 clinic visits. Following the intervention, 324 antibiotic prescriptions were given during 8,442 clinic visits accounting for 38.3 prescriptions per 1,000 clinic visits, a decrease of 9.6%. Patients were similar in age and sex in the pre- and postintervention periods (Table 1).

**Table 1.** Characteristics of Geriatric Clinic Patients and Prescribing Providers

Characteristic	Before the Intervention, No. (%)^a^	After the Intervention, No. (%)^b^	*P* Value (0.66 for Total Prescriptions)
Patient age, median (IQR)	81 (76–86)	81 (76–87)	
**Patient sex**			.12
Male	84 (32)	64 (26)	
Female	179 (68)	185 (74)	
**Provider type for each prescription**			.90
Physician	186 (51)	168 (52)	
Advanced Practice provider	176 (49)	156 (48)	
Encounter type			.89
In-person visit	213 (59)	189 (58)	
Other encounter type	149 (41)	135 (42)	

a N = 362 total systemic antibiotic prescriptions including for respiratory and nonrespiratory infectious indications for 263 patients.

b N = 324 total systemic antibiotic prescriptions including for respiratory and nonrespiratory infectious indications for 249 patients.

Of the 362 antibiotic prescriptions given prior to the intervention, 137 prescriptions were given for acute respiratory tract infection, including 16 for bronchitis, 40 for sinusitis, and 47 for pneumonia. Following the intervention, 112 prescriptions were given over the same duration for acute respiratory tract infections, including 8 for bronchitis, 25 for sinusitis, and 38 for pneumonia (Table 2). The most common indications for the 225 preintervention prescriptions and 212 postintervention prescriptions for antibiotics for nonrespiratory causes were urinary tract infection in 37% and skin and soft-tissue infections in 12%.

**Table 2.** Change in Number and Duration of Antibiotic Prescriptions for Respiratory Infections

Indication	No. of Antibiotic Prescriptions		Duration, Median, (IQR) [Range, Days]		Estimated Change in Duration,	*P* Value
	Before (n=137)	After (n=112)	Before	After	Days (95% CI)^a^	
Pneumonia	47	38	5 (5–10) [1–21]	5 (5–7) [3–10]	−0.9 (−2.0 to 0.2)	.11
Sinusitis	40	25	7 (7–10) [5–14]	7 (5–7) [2–10]	−2.0 (−3.1 to −1.0)	<.001
Pharyngitis	7	11	10 (5–10) [5–10]	5 (5–7) [5–10]	−2.5 (−4.6 to −0.5)	.02
COPD exacerbation	22	23	5 (5–5) [5–14]	5 (5–5) [5–10]	−0.8 (−1.9 to 0.3)	.13
Otitis	5	7	10 (7–10) [7–10]	5 (5–7) [5–7]	−3.2 (−5.2 to −1.3)	.008
Bronchitis	16	8	5 (5,5) [5–14]	5 (5–5) [5–5]	−0.7 (−2.4 to 1.0)	.41
Overall adjusting for indication	137	112	7 (5–10) [1–21]	5 (5–7) [2–10]	−1.3 (−1.9 to −0.8)	<.001

Note. IQR, interquartile range; COPD, chronic obstructive pulmonary disease.

a From linear mixed regression models.

We observed a decrease in the duration of prescribed antibiotics for specific upper respiratory indications (summarized overall in Table 2 and by specific antibiotic in Table 3). The duration of antibiotic use significantly decreased between the pre- and postintervention periods for sinusitis (by 2.0 days), pharyngitis (by 2.5 days), and otitis (by 3.2 days); Table 2. We detected no significant difference in the duration of antibiotic use for pneumonia, COPD exacerbation, or bronchitis (*P* ≥ .11) (Table 2). For sinusitis, the 2 most prescribed antibiotics before the intervention were doxycycline (40%) and amoxicillin-clavulanate (27%) (Table 3). Following the intervention, amoxicillin (48%) and doxycycline (24%) were the 2 most prescribed antibiotics (Table 3). The timing of the prescription (within a clinic encounter vs other such as phone call) was similar between the periods (Table 1).

**Table 3.** Antibiotics Most Commonly Prescribed for Respiratory Indications Before and After the Intervention

Respiratory Syndrome	Antibiotic, by Most Commonly Prescribed	Before the Intervention		After the Intervention	
		No.	Duration (Median, IQR) [Range] Days	No.	Duration (Median, IQR) [Range] Days
Pneumonia	Doxycycline	19	10 (7–10) [5–21]	12	5 (5–7) [5–10]
	Azithromycin	14	5 (5–5) [5–5]	15	5 (5–5) [5–10]
	Levofloxacin	10	4.5 (3–10) [1–10]	3	5 (5–7) [5–7]
	Amox/Clav	3	7 (5–7) [5–7]	4	5 (5–6) [5–7]
	Amoxicillin	1	5 (5–5) [5–5]	4	6 (4–7) [3–7]
Sinusitis	Doxycycline	16	7 (7–10) [5–14]	6	5 (5–7) [2–7]
	Amox/Clav	11	10 (7–10) [5–10]	3	5 (5–10) [5–10]
	Amoxicillin	10	7 (5–7) [5–10]	12	7 (7–7) [5–7]
	Azithromycin	1	5 (5–5) [5–5]	4	5 (5–5) [5–5]
	Other	2	10 (10–10) [10–10]	0	
Pharyngitis	Amoxicillin	5	10 (10–10) [10–10]	5	7 (7–7) [5–10]
	Azithromycin	2	5 (5–5) [5–5]	6	5 (5–5) [5–5]
COPD exacerbation	Azithromycin	16	5 (5–5) [5–5]	18	5 (5–5) [5–5]
	Doxycycline	4	10 (7.5–12) [5–14]	5	10 (5–10) [5–10]
	Amoxicillin	1	10 (10–10) [10–10]	0	
	Levofloxacin	1	7 (7–7) [7–7]	0	
URI, bronchitis, or not otherwise specified	Azithromycin	14	5 (5–5) [5–5]	8	5 (5–5) [5–5]
	Amox/Clav	1	14 (14–14) [14–14]	0	
	Doxycycline	1	7 (7–7) [7–7]	0	

Note. IQR, interquartile range; COPD, chronic obstructive pulmonary disease; URI, upper respiratory infection; Amox/Clav, amoxicillin–clavulanate.

**Fig. 1.**
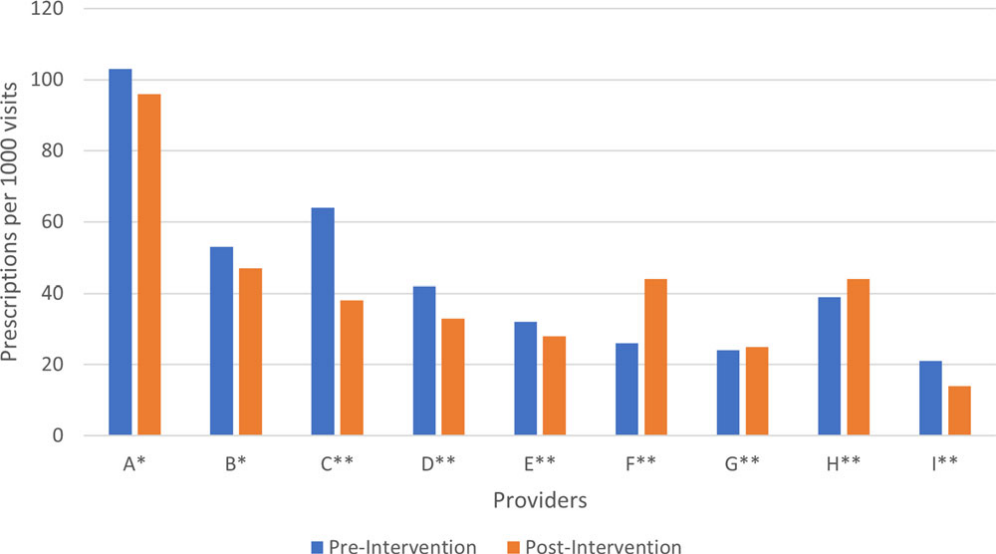
Pre- and Post-Intervention Antibiotic Prescribing rate per 1000 visits, for individual providers descriptions of labels. Note. *NP, nurse practitioner and **MD, medical doctor.

Lastly, we explored whether the change in prescriptions was driven by individual prescribers. Of the 362 antibiotic prescriptions before the intervention, 266 (72%) were given by 4 providers (Fig. 1). Following the intervention, the number of prescriptions for these 4 providers decreased to 195 (60%) of the total prescriptions given during this period. The change in antibiotic prescriptions was similar among both physicians and advanced practice providers.

## Discussion

In this pilot-phase quality improvement intervention, we first demonstrated the prevalence and types of inappropriate antibiotic prescribing in one of our clinics. We were able to accurately quantify the outpatient antibiotic use with the help of our newly created electronic report. By focusing on specific aspects of antibiotic prescribing, we were then able to decrease the use of broad-spectrum antibiotics as well as total antibiotic use (ie, number of prescriptions, duration of antibiotics) for certain acute respiratory conditions through a multistep intervention. Using a low-cost, less resource-intense approach, we were able to demonstrate a modest decrease in antibiotic use (10%), which is similar to that of a prior study by March-Lopez et al,^20^ who reported a decrease of 17%. Another recent large study from the Veterans’ Affairs health system showed an overall annual reduction of 3.9% from 2011–2018 across 1,200 outpatient clinics.^21^

One of the biggest challenges in implementing outpatient antimicrobial stewardship programs is the capability to appropriately track data of outpatient antibiotic use. Most prior studies have used large administrative claims database,^5^ and evaluating institution and clinic-level data has been challenging. Here, we were able to utilize report-building tools in the electronic health record system (Epic software) to track all antibiotic prescriptions generated in the outpatient setting. This allowed us to measure the impact of our intervention on the prescription, indication, and duration of antibiotics.

Next, although antibiotic stewardship programs are most active in the inpatient setting, the most effective type of intervention for decreasing outpatient prescriptions is not known. Multiple different approaches have been tried to improve antibiotic prescribing including educational approaches, behavioral approaches, and technological support.^16–18^ Multifaceted antimicrobial stewardship interventions have shown to be successful in decreasing antibiotic consumption and improving overall antibiotic use.^20^ We implemented multiple low-cost interventions including an interactive session with providers, use of posters, and antibiotic order panels in the electronic record system. A flexible approach that can be tailored to individual clinic and practice settings has a better chance of being successful.

Our intervention was able to both decrease antibiotic prescriptions and better match the antibiotic and duration to guideline-based recommendations. In a prior study, we demonstrated that 81% of antibiotic prescriptions for acute sinusitis and 48% of antibiotic prescriptions for pharyngitis were inappropriate.^22^ The types of prescribing errors differed between infections. For sinusitis, lack of an indication for antibiotics, and excessive duration of antibiotics were common errors. Excessive antibiotic duration was noted in ∼50% of sinusitis patients. In other studies, the highest rates of unnecessary prescribing have been noted for acute bronchitis, acute sinusitis, and viral upper respiratory infections,^6,13,23^ where azithromycin, fluoroquinolones, and amoxicillin-clavulanate comprise a majority of the inappropriate antibiotic prescriptions.^7,8^ Consistent with these studies, azithromycin was the most prescribed antibiotic in our population as well. Before the intervention, the geriatric clinic had higher use of narrower-spectrum antibiotics, particularly doxycycline, compared to previous studies^5,6^; however, the intervention was able to limit the duration of antibiotics for those already prescribed the appropriate agent.

Our intervention also targeted the prescriber. Prior research has shown that mid- or late-career providers and providers with high patient volumes tend to have a greater proportion of antibiotic prescriptions.^13^ However, with our intervention, nearly all providers had a decrease in the number of total prescriptions (Fig. 1). As we were unable to provide a denominator for the number of visits presenting with respiratory symptoms, we cannot rule out that changes in prescribing may represent an increase or decrease in the number of patients presenting with symptoms and thus, differences in opportunities to implement the recommendations. Furthermore, the rate of antibiotic prescribing and the response to the intervention was similar among both advanced practice providers and physicians.

As noted in prior studies, antibiotic prescriptions are frequently given without a face-to face encounter.^8^ In our study, 41% of prescriptions were given outside a clinic encounter. Although these prescriptions may have followed an in-person or telehealth visit after results of a chest radiograph or blood work, others may have prescribed over the phone with limited patient evaluation. Many of these antibiotic practices are influenced by local prescribing practices and patient expectations, and it is important to understand the local culture to affect meaningful change.^24^

The strengths of our project include our stepwise, multicomponent approach, partnership with a specific clinic, and development of an electronic reporting tool. However, our reporting tool may have missed antibiotic durations for some antibiotics (particularly azithromycin), caused some underestimation of antibiotic prescriptions if alternate methods of prescribing, for example written or telephone prescriptions were used. Furthermore, there may be a discrepancy between antibiotic prescription and actual antibiotic use. Additional limitations include our inability to evaluate change in prescriptions after each intervention and overlap between a couple of the interventions (patient education poster, viral prescription pads) with the postintervention data collection period.

In summary, low-cost interventions can be successfully used to improve antibiotic prescriptions to decrease overall outpatient antibiotic use. Longer-term sustainability of these interventions and scalability to other clinics and infectious syndromes should be evaluated.

## Supplementary material

For supplementary material accompanying this paper visit https://doi.org/10.1017/ash.2021.238. click here to view supplementary material
