# Supplementary material for: Impact of a pilot multimodal intervention to decrease antibiotic use for respiratory infections in a geriatric clinic
Source: Antimicrob Steward Healthc Epidemiol. 2022 Jan 10;2(1):e1. doi: 10.1017/ash.2021.238 (PMC9614947; doi:10.1017/ash.2021.238)
Supplement: Supplementary file 1 [file S2732494X21002382sup001.docx]

**Table 1: Number of total prescriptions and prescriptions per 1000 visits per provider (pre and post-intervention)**

| Provider ID | Number of Prescriptions (pre-intervention) | Number of visits (pre-intervention) | Prescriptions per 1000 visits | Number of prescriptions (post-intervention) | Number of visits (post-intervention) | Prescriptions per 1000 visits (post-intervention) |
| --- | --- | --- | --- | --- | --- | --- |
| Provider A | 112 | 1069 | 105 | 87 | 903 | 96 |
| Provider B | 72 | 1337 | 54 | 58 | 1226 | 47 |
| Provider C | 51 | 732 | 70 | 25 | 657 | 38 |
| Provider D | 45 | 870 | 52 | 25 | 761 | 33 |
| Provider E | 19 | 596 | 32 | 16 | 566 | 28 |
| Provider F | 17 | 684 | 25 | 28 | 636 | 44 |
| Provider G | 18 | 667 | 27 | 18 | 706 | 25 |
| Provider H | 15 | 361 | 42 | 16 | 359 | 44 |
| Provider I | 8 | 374 | 21 | 5 | 368 | 14 |

**Table 2: Institutional Antibiotic Duration Recommendations**

| **Indication** | **Goal Duration of therapy (days)** |
| --- | --- |
| Acute exacerbation of chronic bronchitis | 5 |
| Pneumonia | 5 |
| Sinusitis | 5 |
| Skin and skin structure infection | 5 |
| Otitis | 7 |
| Urinary tract infection | 7 |

**Figure 1: Use of different antibiotics: pre and post-intervention**

**Image 1: Viral Prescription Pad distributed in clinic**

**
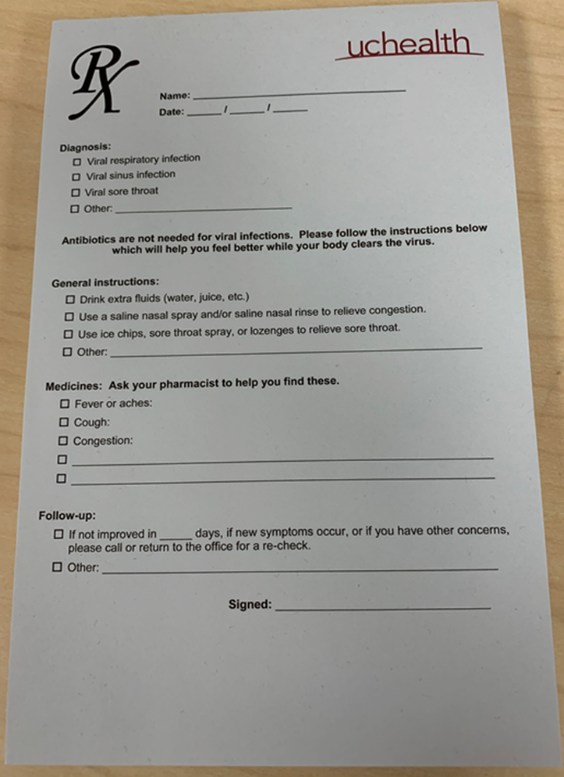
**

**Image 2: Poster in patient waiting area**

**
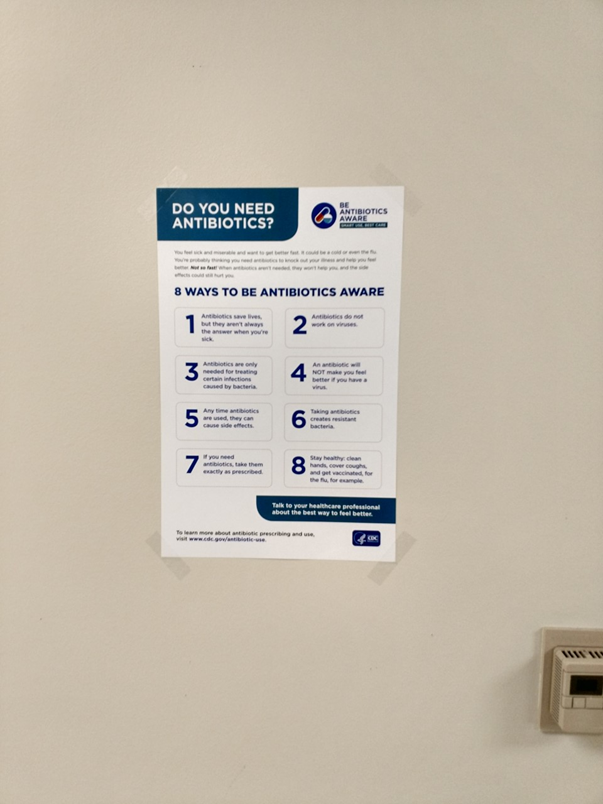
**

**Image 3: Poster in patient waiting area**

**
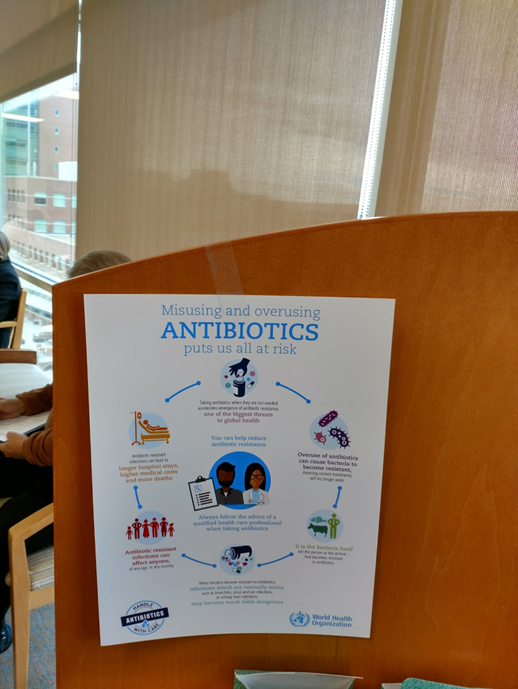
**
